# Diagnostic Accuracy of Procalcitonin, Neutrophil-to-Lymphocyte Ratio, and C-Reactive Protein in Detection of Bacterial Infections and Prediction of Outcome in Nonneutropenic Febrile Patients with Lung Malignancy

**DOI:** 10.1155/2020/2192378

**Published:** 2020-08-25

**Authors:** Shanshan Ding, Jun Ma, Xingguo Song, Xiaohan Dong, Li Xie, Xianrang Song, Lisheng Liu

**Affiliations:** ^1^Department of Clinical Laboratory, Shandong Cancer Hospital and Institute, Shandong First Medical University and Shandong Academy of Medical Sciences, Jinan, Shandong, China; ^2^Department of Foreign Affairs for Scientific Research, Shandong Cancer Hospital and Institute, Shandong First Medical University and Shandong Academy of Medical Sciences, Jinan, Shandong, China; ^3^Shandong Provincial Key Laboratory of Radiation Oncology, Shandong Cancer Hospital and Institute, Shandong First Medical University and Shandong Academy of Medical Sciences, Jinan, Shandong, China

## Abstract

**Background:**

Procalcitonin (PCT), C-reactive protein (CRP), and neutrophil-to-lymphocyte ratio (NLR) have emerged as important markers of inflammation, and these markers, especially PCT and CRP, have been studied in patients with neutropenia. This study was designed to evaluate their value in differentiating infectious fever from tumor fever (TF) and to investigate their role in assessing outcomes in nonneutropenic lung cancer patients (NNLCPs).

**Methods:**

This retrospective clinical study included 588 febrile NNLCPs between January 2019 and December 2019. The levels of PCT, CRP, and conventional inflammatory markers, including white blood cells (WBC) and neutrophils (NEU), were measured. NLR was defined as the ratio of the absolute neutrophil count to the absolute lymphocyte count. Patients' clinical and bacteriological data were recorded.

**Results:**

This study included 311 NNLCPs with bacterial infections and 277 with TF. Inflammatory markers such as PCT, CRP, WBC, and NEU levels and NLR were significantly higher in patients with bacterial infections than in those with TF (*p* < 0.0001). However, PCT level was the best predictor of bacterial infections, with an area under the curve (AUC) of 0.874, followed by CRP level (AUC = 0.855) and NLR (AUC = 0.792) (*p* < 0.0001). Additionally, PCT level was significantly elevated in patients with bacterial infections with progressive disease after radiotherapy and chemotherapy (*p* < 0.01).

**Conclusions:**

The present study demonstrated the superiority of PCT over CRP and NLR in the diagnosis of febrile patients with bacterial infections. Additionally, PCT can be used to assess the clinical outcomes and cancer progression in NNLCPs.

## 1. Introduction

Patients with lung cancer are susceptible to bacterial infections owing to their compromised immune system [1], leading to high morbidity and mortality [2, 3]. Although neutropenia is known to be a major risk factor for infections [4], few studies have evaluated nonneutropenic lung cancer patients (NNLCPs). In NNLCPs, fever may be caused by bacterial infections or by tumor fever (TF) [5, 6]. Considering that immediate empirical antibiotic therapy has been widely recommended for febrile patients [1, 5], patients with nonbacterial infections might be exposed to unnecessary antibiotics. This excessive use of antibiotics can result in toxicity, bacterial resistance, increased patient mortality, and significant medical costs [5, 7]. Thus, a differential diagnosis of fever at the initial stage of therapy is important [8].

In such cases, bacterial infections in NNLCPs should be identified to provide appropriate and effective treatment. Most bacterial infections can be diagnosed based on clinical symptoms, inflammatory markers, radiological imaging, and microbiological data [1, 9]. Currently, conventional biomarkers such as white blood cell (WBC) count, neutrophil (NEU) count, erythrocyte sedimentation rate, and clinical signs and symptoms are not sufficiently sensitive and specific to guide treatment decisions for NNLCPs with fever [10–12]. Moreover, as the gold standard for bacterial infections, microbiological cultures have certain disadvantages, including diagnostic delays (as a result of culture methods), suboptimal sensitivity (blood cultures), and low specificity due to contamination (sputum cultures) [10, 12, 13]. Therefore, reliable specific and sensitive markers are needed for NNLCPs with fever.

Several inflammatory markers have been evaluated for the diagnosis of infections. Procalcitonin (PCT), a precursor of the hormone calcitonin [5], has emerged as a promising marker for bacterial infections in various diseases [14–18]. Some studies have shown that PCT might be superior to commonly used clinical variables and other laboratory tests in the diagnosis of infections [19–22]. Most importantly, PCT level appears to correlate with the extent and severity of infection [23–25]. C-reactive protein (CRP), another marker for diagnosing infections, is produced primarily by hepatocytes in response to infection [2]. Several studies have also indicated that PCT and CRP levels can be used to distinguish infectious fever from TF in patients with febrile neutropenia [5, 26, 27]. Neutrophil-to-lymphocyte ratio (NLR), the easily measured, reproducible, and inexpensive marker of disease [28], has been reported to be a diagnostic marker for cancer [29–33]. Moreover, it is indicative of an impaired cell-mediated immunity associated with systemic inflammation [28], thus influenced by many conditions like metabolic disorder including diabetes mellitus and obesity [34]. It has been reported that an increased level of NLR in peripheral blood can predict the development of type 2 diabetes in morbid obese patients with high sensitivity and specificity. Thus, it has attracted attention in recent years. In addition, previous research had reported platelet count and platelet indices are the indicators of platelet function and activation, which have been evaluated for diagnosis of liver fibrosis [35]. Chronic inflammatory process is associated with neoplastic transformation; thus altered platelet count and platelet indices might serve as potential biomarkers for the diagnosis and prognosis of cancer [36]. In summary, many studies have evaluated the diagnostic utility of inflammatory markers in various diseases and have particularly assessed the role of PCT and CRP in patients with febrile neutropenia. However, their value as a diagnostic tool in NNLCPs needs further study.

Inflammation is an important marker of outcomes in cancer. Accumulating evidence has been found regarding the role of inflammatory markers in cancer development and progression [5, 28, 37–39]. In a study that evaluated PCT level in patients with solid tumors and no evidence of infection, higher PCT level was found in patients with extensively metastatic cancer [40]. Thus, PCT level can be considered a predictor of clinical outcomes in patients with cancer without bacterial infections. Similarly, the CRP level has been studied for its predictive and prognostic value in various human malignancies [41, 42]. Some studies have found that PCT and CRP levels are associated with tumor stage [1, 40, 43] and that their levels are higher in patients with advanced tumors than in patients with early-stage cancer [5, 44, 45]. At the same time, the prognostic role of NLR has been documented in various cancers [46–48]. For example, in patients with breast cancer, an elevated NLR typically indicates shorter disease-free survival and overall survival [49]. In summary, recent studies have confirmed a relationship between inflammatory markers and poor prognosis. To the best of our knowledge, however, few studies have investigated the role of these markers in assessing outcomes and in cancer progression in NNLCPs with bacterial infections.

Thus, in the present study, we first evaluated the diagnostic value of PCT, CRP, and NLR compared with conventional biomarkers such as WBC and NEU counts in determining bacterial infections in febrile NNLCPs. Next, we assessed their usefulness in predicting clinical outcomes and cancer progression in NNLCPs. The study findings should be validated in future studies with a higher number of patients.

## 2. Materials and Methods

### 2.1. Patient Eligibility and Classification

In this retrospective clinical observational study, we included 588 NNLCPs with fever who were admitted to Shandong Cancer Hospital and Institute from January 2019 to December 2019. Clinical characteristics were collected from the patients' electronic medical records and the Ruimei laboratory information system version 6.0 (rmlis, Huangpu District, Shanghai, China).

Figure S1 shows 588 patients with nonneutropenic fever who were divided into two groups on the basis of their clinical, microbiological, and radiological data. The bacteria-infected group comprised 311 patients with microbiologically documented infection (MDI), which was defined as fever with positive bacterial cultures from specimens. The TF group comprised 277 patients without microbiological confirmation, radiological evidence, or clinically documented infections. Patients who met the following criteria were enrolled in this study: axillary temperature >37.5°C and absolute neutrophil count not lower than 0.5 × 10^9^/L. Hematological parameters and inflammatory mediators such as PCT and CRP levels were determined within 48 h of the onset of fever, and bacterial cultures were taken. Prior to the start of antibiotic treatment, all measurements were performed. Patient diagnoses were confirmed via pathological examination. At the same time, patients with certain influential factors such as immune system disease, metabolic diseases, cardiovascular disease, the use of corticosteroid drugs, and the infection of other pathogenic microorganisms (e.g., virus, fungus, and parasite) were excluded.

### 2.2. Bacterial Culture and Identification

Bacterial cultures from specimens such as blood, mid-stream urine, sputum, and drainage fluid were prepared and analyzed. For blood cultures, blood samples were collected several times during the fever cycle. If the culture result was confirmed to be positive within 24 h, two or more blood samples were collected for analysis. Sputum and mid-stream urine were collected in the early morning. All specimens were collected before antibiotic treatment and were transferred to the laboratory within 1 h after collection. The results of bacterial cultures were considered as the gold standard for the diagnosis of infection. After bacterial cultures, biotypes were identified using the Biomerieux ATB Expression system, and biotypes that were commonly contaminated by a single culture and could not be excluded were eliminated. All procedures were conducted in accordance with the manufacturers' instructions and the BC standards of the Clinical Laboratory Standards Institute [50].

### 2.3. Laboratory Measurement of Inflammatory Markers

The serum PCT level was determined by a quantitative electrochemiluminescence immunometric assay using a COBAS E602 analyzer (Roche Diagnostics GmbH, Mannheim, Germany), with level of <0.05 ng/mL considered normal.

Plasma CRP level was measured by immunoturbidimetry assay using Beckman Coulter analyzer AU5800 (Beckman Coulter, Brea, CA, USA). The reference value provided by the manufacturer was <10 mg/L.

For hematological studies, we collected whole blood in sterile tubes containing K2-ethylenediaminetetraacetic acid, and blood counts were estimated using automatic hematological analyzer Sysmex XN9000 (Sysmex Corporation, Kobe, Japan). Total peripheral leukocyte, lymphocyte, monocyte, and neutrophil levels were also recorded. NLR was defined as the ratio of the absolute neutrophil count to the absolute lymphocyte count. All tests were performed according to the manufacturer's instructions.

### 2.4. Statistical Analysis

Statistical analysis was performed using SPSS version 22.0 statistical software (SPSS, Chicago, IL, USA) and GraphPad Prism version 6.0 (GraphPad Software, San Diego, CA, USA). For comparisons of nonnormal continuous variables, the Mann–Whitney *U* test was used for two independent samples. Spearman's correlation was used to compare the correlation between two variables. Receiver operating characteristic (ROC) curve analysis was implemented to evaluate the effects of markers for predicting bacterial infections. The optimal cut-off points were determined using the Youden index. Data were presented as the median ± interquartile range (range, minimum–maximum). Significance was established at *p* < 0.05.

## 3. Results

### 3.1. Patient Characteristics and Microbiology Results

A total of 588 NNLCPs were analyzed in this study (Figure S1). The clinical and demographic characteristics of the bacteria-infected group (*N* = 311) and TF group (*N* = 277) are shown in Table 1, including age, sex, pathological type of cancer, tumor stage, and hematological parameters. The median age was 65 (range, 35–87) years in the bacteria-infected group and 64 (range, 28–84) years in the TF group. Regarding the causative pathogens, seven patients were identified by MDI as having simultaneous infection with two types of bacteria; 318 cases in the bacteria-infected group had positive bacterial cultures, including 44 (13.8%) cases of Gram-positive bacteria and 274 (86.2%) cases of Gram-negative bacteria. No significant differences were found between Gram-positive bacteria and Gram-negative bacteria in PCT, CRP, WBC, NEU, and NLR levels (Table S1). Among Gram-positive bacteria, *Staphylococcus aureus* was the most common species (6.3%), followed by *Streptococcus pneumoniae* (4.7%). Among Gram-negative bacteria, the most frequently identified species were *Klebsiella pneumoniae* (21.4%) and *Haemophilus influenzae* (18.2%), followed by *Pseudomonas aeruginosa* (13.9%), *Escherichia coli* (9.4%), *Acinetobacter baumannii* (5.4%), and *Moraxella catarrhalis* (4.4%). Names of all the bacterial taxa are shown in Table 1.

### 3.2. NLR Had a Greater Capacity for the Diagnosis of Bacterial Infections than WBC and NEU in NNLCPs with Fever

**Figure 1:**
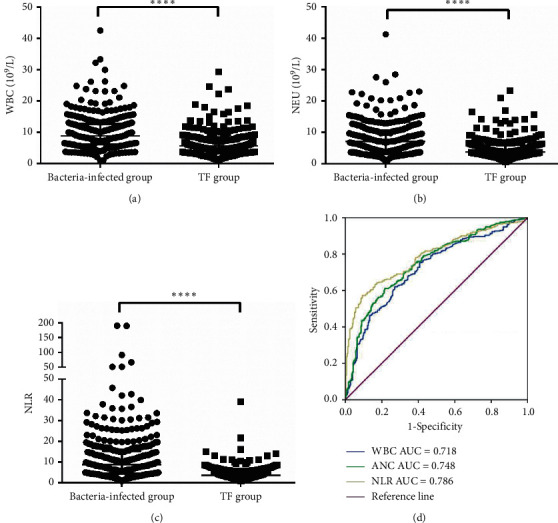
Inflammatory markers (WBC, NEU and NLR) between bacteria-infected group (*N* = 311) and TF group (*N* = 277) in NNLCPs with fever. The differences of WBC (a) and NEU (b) levels and NLR (c) between bacteria-infected group (*N* = 311) and TF group (*N* = 277). The horizontal lines represented the median. Statistical significance was calculated with Mann–Whitney test. (d) ROC curves of the sensitivity and specificity for WBC, NEU, and NLR. ROC curves were calculated predicting the absence or presentation of infection in febrile NNLCPs. ^*∗∗∗∗*^*p* < 0.0001; WBC: white blood cell; NEU: neutrophil; NLR: neutrophil-to-lymphocyte ratio; TF: tumor fever; AUC: area under the ROC curve; NNLCPs: nonneutropenic lung cancer patients.

We first confirmed whether WBC and NEU levels and NLR could be used in the diagnosis of bacterial infections for NNLCPs as shown in the bacteria-infected and TF groups (Figure S1).  Figure 1(a) shows that patients with MDI had a higher median WBC count (8.87 × 10^9^/L) than with NNLCPs without bacterial infections (5.57 × 10^9^/L) (*p* < 0.0001). A significant difference in the NEU level was found between the bacteria-infected (7.15 × 10^9^/L) and TF groups (3.82 × 10^9^/L) (*p* < 0.0001) (Figure 1(b)). As shown in Figure 1(c), patients with MDI had higher median NLR values (8.70) than patients with TF (3.63) (*p* < 0.0001).

To determine the diagnostic accuracy of WBC, NEU, and NLR in bacterial infections, we constructed ROC curves. The analysis revealed that in NNLCPs with MDI, WBC had a discriminative power, with an AUC of 0.718 (95% confidence interval [CI] 0.677–0.759), sensitivity of 62.1%, and specificity of 72.9% compared with the TF group. For NEU, the AUC was 0.748 (95% CI 0.709–0.787), with 61.1% sensitivity and 78.3% specificity. Meanwhile, the ROC curves indicated that NLR had an AUC of 0.786 (95% CI 0.749–0.823), with 57.2% sensitivity and 90.6% specificity (Figure 1(d)).

### 3.3. Comparison of PCT, CRP, and NLR for Bacterial Infections in Febrile NNLCPs

In order to achieve better diagnostic accuracy, the classic inflammatory markers PCT and CRP were used. A total of 125 eligible patients were screened for their PCT and CRP levels. 69 patients were assigned to the bacteria-infected subgroup A, and the remaining 56 were assigned to the TF subgroup A (Figure S1).

**Figure 2:**
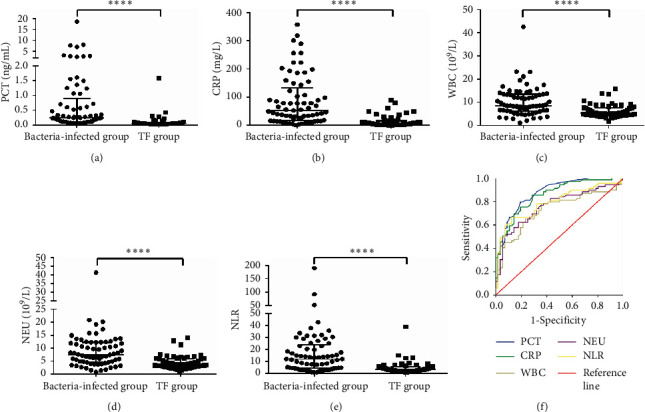
Inflammatory markers (PCT, CRP, WBC, NEU, and NLR) between bacteria-infected subgroup A (*N* = 69) and TF subgroup A (*N* = 56) in febrile NNLCPs. The differences of PCT (a), CRP (b), WBC (c) and NEU (d) levels, and NLR (e) between bacteria-infected subgroup A (*N* = 69) and TF subgroup A (*N* = 56). The horizontal lines represented the median. Statistical significance was calculated with Mann–Whitney test. (f) ROC curves analysis for PCT, CRP, WBC, NEU, and NLR in subgroup A of NNLCPs. ^*∗∗∗∗*^*p* < 0.0001; PCT: procalcitonin; CRP: C-reactive protein; WBC: white blood cell; NEU: neutrophil; NLR: neutrophil-to-lymphocyte ratio; TF: tumor fever; AUC: area under the ROC curve; NNLCPs: nonneutropenic lung cancer patients.

The median level and range of blood inflammatory markers (PCT, CRP, WBC, NEU, and NLR) in the two subgroups are shown in Table 2. These markers differed between the subgroups. There was a markedly significant difference in the median PCT level between bacteria-infected subgroup A (0.24 ng/mL) and TF subgroup A (0.05 ng/mL) (*p* < 0.0001) (Figure 2(a)). Similarly, the median CRP level of bacteria-infected subgroup A (53.2 mg/L) was higher than that of TF subgroup A (6.3 mg/L) (*p* < 0.0001) (Figure 2(b)). Furthermore, bacteria-infected patients had higher median WBC and NEU levels (8.49 × 10^9^/L and 7.39 × 10^9^/L, respectively) than patients with nonbacterial infections (5.24 × 10^9^/L and 3.56 × 10^9^/L, respectively, *p* < 0.0001) (Figures 2(c) and 2(d)).  Figure 2(e) shows that the patients who had positive bacterial cultures had higher median NLR values (12.15) than the patients with TF with nonneutropenic lung cancer (3.27) (*p* < 0.0001).

Table 3 shows Spearman's correlations among the test parameters in bacteria-infected subgroup A. As expected, the circulating markers of inflammation tended to be significantly correlated. However, WBC level was not correlated with PCT and CRP levels (Spearman's *r* = 0.222, *p* = 0.067 and *r* = 0.176, *p* = 0.148, respectively).

To evaluate the discriminative power of inflammatory markers to predict infections in subgroup A of nonneutropenic lung cancer, we constructed ROC curves.  Table 4 shows the evaluation of AUC, sensitivity, specificity, negative predictive value, positive predictive value, and the Youden index. These cut-off values were calculated based on the best sensitivity and specificity (Youden index), which were found to have the highest diagnostic value for predicting bacterial infections.  Figure 2(f) shows that of the inflammatory markers, PCT was the best predictor, with an AUC of 0.874 (95% CI 0.813–0.935), followed by CRP, with an AUC of 0.855 (95% CI 0.790–0.919). On comparison of the patients with MDI with the noninfected patients, the ROC curves of WBC, NEU, and NLR showed that the AUC was 0.729 (95% CI 0.639–0.818), 0.761 (95% CI 0.676–0.846), and 0.792 (95% CI 0.712–0.872), respectively.

### 3.4. PCT Level Was Elevated in NNLCPs with Bacterial Infections and Progressive Disease (PD)

To study blood inflammatory markers for clinical outcome assessment in NNLCPs, we analyzed the relationship between response to therapy and the levels of inflammatory markers in bacteria-infected subgroup A and TF subgroup A.

**Figure 3:**
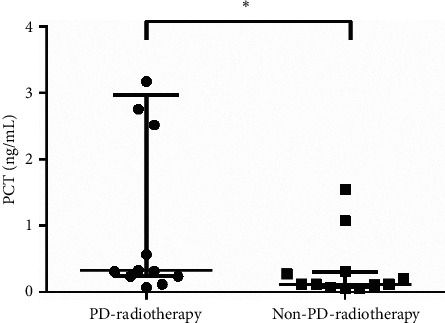
Correlation between PCT level and clinical response to radiotherapy in bacteria-infected subgroup A. Before microbiologically documented infections, patients underwent a series of clinical anticancer treatments. PD-radiotherapy (response to radiotherapy was PD) patients (*n* = 13) had higher PCT level than non-PD-radiotherapy (response to radiotherapy was SD and PR) patients (*n* = 12) in bacteria-infected subgroup A. Statistical significance was calculated with Mann–Whitney test. ^*∗*^*p* < 0.05; PCT: procalcitonin; PR: partial response; SD: stable disease; PD: progressive disease.

As shown in Table 5, patients with MDI were treated with various therapies, including radiotherapy [30], chemotherapy [15], surgery [19], and others (targeted combination, conservative treatment, or suspension therapy) [5]. Twenty-five patients in bacteria-infected subgroup A received radiotherapy; of these, 13 developed PD, 11 patients had stable disease (SD), and 1 showed partial response (PR). In terms of response to radiotherapy, patients with PD-radiotherapy (response to radiotherapy was PD) had higher PCT level than patients with non-PD-radiotherapy (response to radiotherapy was SD and PR) (*p* = 0.0116) (Figure 3). Remarkably, no differences were found between patients with PD-radiotherapy and those with non-PD-radiotherapy in CRP level and NLR (data not shown). However, the CRP level and NLR were higher in the PD-radiotherapy group than in the non-PD-radiotherapy group. Three patients had PD and 12 had SD after chemotherapy in bacteria-infected subgroup A. Owing to the small sample size, statistical differences were not analyzed.

Next, we evaluated the inflammatory marker levels for clinical response assessment in TF subgroup A. Detailed treatment methods and outcomes of this group are shown in Table 5. Of the 27 patients who received radiotherapy, 2 had PD, 13 had SD, and 4 had PR, whereas 8 had no clear efficacy evaluation according to Response Evaluation Criteria In Solid Tumors guidelines [51]. Of the 21 patients who underwent chemotherapy, except those for whom the efficacy was not clear, 10 had SD and 3 showed PR. However, statistical differences between the levels of inflammatory markers and response to therapy were not analyzed owing to the small sample size.

### 3.5. PCT Can Be Used to Assess Clinical Outcomes for NNLCPs with Bacterial Infections

Collectively, based on the existing studies, a correlation between PCT level and clinical response was identified and then expanded to other patients for future validation. Because of the low number of PCT measurements conducted in the TF group, no further research was conducted. Therefore, we screened all patients with MDI tested for PCT and analyzed the relationship between outcomes and inflammatory markers in bacterial-infected group B (*N* = 191) (Figure S1).

Given the good correlation between PCT and the other inflammatory markers described above, we classified patients according to PCT values measured on presentation with MDI in bacteria-infected subgroup B. As shown in Table 6, categorization according to PCT level revealed clear distinctions with respect to WBC and NEU levels and NLR, all of which were significantly higher in the high PCT group than in the low PCT group.

**Figure 4:**
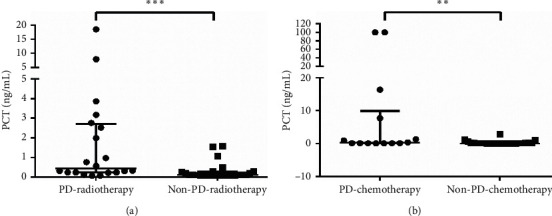
Correlation between PCT level and clinical response in bacteria-infected subgroup B. (a) PCT level expressed higher in PD-radiotherapy (response to radiotherapy was PD) (*n* = 20) than that in non-PD-radiotherapy (response to radiotherapy was SD and PR) (*n* = 25) of bacteria-infected subgroup B. (b) For patients treated with chemotherapy, PCT level of patients with PD (PD-chemotherapy) (*n* = 14) was higher than non-PD-chemotherapy (response to chemotherapy was SD and PR) (*n* = 24) patients in bacteria-infected subgroup B. Statistical significance was calculated with Mann–Whitney test. ^*∗∗*^*p* < 0.01; ^*∗∗∗*^*p* < 0.001; PCT: procalcitonin; PR: partial response; SD: stable disease; PD: progressive disease.

As described in Table 7, of 191 patients with MDI, 62 received radiotherapy, 50 received chemotherapy, 66 underwent surgical treatment, and 13 were referred mainly to targeted combination therapy, conservative treatment, or suspension therapy. Of the 62 patients receiving radiotherapy, 45 had a clear efficacy evaluation. Of these, 20 had PD, 23 had SD, and 2 had PR. In particular, PCT level was higher in the PD-radiotherapy group than in the non-PD-radiotherapy group of bacteria-infected subgroup B (*p* = 0.0002) (Figure 4(a)).

Of the 38 patients who received chemotherapy and had clear efficacy, 14 had PD, 21 had SD, and 3 showed PR. According to the outcomes, PCT level of patients with lung cancer with PD (PD-chemotherapy) was higher than that of patients with non-PD-chemotherapy (the response to chemotherapy was SD and PR) in bacteria-infected subgroup B (*p* = 0.0057) (Figure 4(b)).

Although other inflammatory markers were not significantly different between patients with PD and non-PD following Mann–Whitney tests analysis (data not shown), their levels tended to be higher in the PD group than in the non-PD group. Collectively, our data support our conclusion that PCT level can be used to assess the outcomes of bacteria-infected NNLCPs.

## 4. Discussion

It is estimated that almost 50% of patients with solid tumors and 60% of patients with hematological malignancies die from infectious complications [45], in which neutropenia is an important risk factor for infection [4]. However, few studies have evaluated the role of inflammatory markers in predicting infections in febrile NNLCPs.

In our study, the febrile episodes were retrospectively classified into bacterial infections with MDI and TF. Our data demonstrated that PCT, CRP, WBC, and NEU levels and NLR were predictive of infectious fever in NNLCPs. There were significant differences in these inflammatory markers between the nonneutropenic patients with infection and those with TF. Our results are consistent with those of previous reports [26, 27], which could be helpful in identifying the cause of fever in NNLCPs and guide appropriate and effective antibiotic treatment [52]. To evaluate the discriminative power of inflammatory markers to distinguish between infectious fever and TF, we plotted ROC curves. The sensitivity, specificity, and best cut-off values for clinical use were also presented. Compared with CRP level, NLR, PCT level could more effectively distinguish between TF and infectious fever. ROC analysis indicated that PCT level was the best predictor for bacterial infections in NNLCPs, demonstrating the highest AUC, followed by CRP level and NLR. Although conventional blood inflammatory parameters such as WBC and NEU levels are easily measured and inexpensive [28], they have limited discriminative power and utility for predicting infections. These findings are consistent with those of previous studies. It has been reported that PCT had the better discriminative power than CRP to differentiate not only TF from LBI (localized bacterial infection) (AUC was 0.840 for PCT and 0.786 for CRP) [1], but also bacteremia from nonbacteremia in cancer patients with febrile neutropenia (AUC was 0.748 for PCT and 0.655 for CRP) [53], as well as bacterial infection from other causes of fever in pediatric oncology patients with febrile neutropenia (AUC was 0.769 for PCT and 0.596 for CRP) [54].

However, some reports have indicated that the usefulness of these markers in the diagnosis of infectious diseases in clinical practice is disputable. One study retrospectively evaluated the value of PCT level for infectious fever and TF in febrile episodes in patients with cancer [55]. Although PCT level in the former group appeared to be higher than that in the latter group, this difference was not statistically confirmed [56]. It was also reported that neither the PCT test nor the CRP test was used to diagnose the cause of febrile neutropenia, except in cases of severe sepsis [57, 58]. It is possible that these inconsistent findings could be attributed to the heterogeneity of the enrolled patients: their study mainly focused on hematological malignancies with febrile neutropenia, whereas we aimed to evaluate the diagnostic role of inflammatory markers for infections in febrile NNLCPs. On the other hand, the fact that the patients were divided into two groups based on the presence or absence of bacterial infections with MDI in our study, in contrast to that in other studies, represents an important limitation of our study.

As mentioned above, the use of inflammatory markers as biomarkers for diagnosing bacterial infections has been previously discussed [14–18]. Studies have shown that these markers are helpful in distinguishing various types of infections. For example, PCT and CRP levels were higher in patients with bacterial pneumonia infected with Gram-negative bacteria than in those infected with Gram-positive bacteria; this finding could aid clinicians in determining the appropriate use of antibiotics as a supplementary means [2]. Similarly, some authors have found higher PCT level in cases of Gram-negative bacterial infections at the onset of fever compared with those in cases of Gram-positive bacterial infections or TF, whereas the other biomarkers did not show significance in children and adult patients with febrile neutropenia [52, 59, 60]. The lipopolysaccharide (LPS) structure of the outer membrane of Gram-negative bacteria may be responsible for this phenomenon. The endotoxin LPS can not only induce inflammatory response and cytokine production, but also elevate the expression of PCT at the mRNA and protein levels dependent on nuclear transcription factor *κ*B (NF-*κ*B) [61]. Unfortunately, among Gram-negative and Gram-positive cultures isolated in our study, no significant differences were observed between the two types of bacteria in inflammatory markers in NNLCPs, perhaps due to the different patient profiles or inclusion and exclusion criteria.

As far as clinical outcomes are concerned, little is currently known about the relationship between inflammatory markers and cancer progression in NNLCPs with bacterial infections. Previous reports focused on the value in assessing the efficacy of antibiotic treatment in patients with bacterial infections [45]. It was shown that inflammatory marker levels after fever onset were significantly lower than those at fever onset in patients who exhibited a response to antibiotic therapy [5]. Thus, we analyzed the value of inflammatory marker levels for clinical response assessment. In the study of patients with bacterial infections, for PCT, the outcome evaluation ability considerably improved when we expanded the sample size to 191. The results showed that PCT could predict disease progression for NNLCPs with infections. In contrast, neither CRP level nor NLR had a correlation with clinical outcomes, nor did NEU or WBC levels. For NEU and WBC levels, low reliability and susceptibility limited the study [62]. For CRP level, the small sample size was the limiting factor; however, its level was higher in nonsurvivors than in survivors. Therefore, we do not deny the value of CRP level in outcome assessments in an expanded cohort. For NLR, neutrophils and lymphocytes can be affected by various factors, including bacterial infections, radiochemotherapy, and autoimmunity [28], which hinder outcome assessments in patients with inflammation. With regard to noninfected patients, an analysis was not performed due to the low number of available studies. However, our data demonstrated that patients with TF mainly had SD after anticancer treatments, whereas patients with febrile infections had poorer clinical treatment responses, which strengthens the usefulness of inflammatory markers in outcome assessment in NNLCPs.

Additionally, considering that inflammatory markers can predict cancer progression [5, 31–33], PCT and CRP levels and NLR in patients with TF were compared by lung cancer stages. Our results showed no significant differences, but the values tended to be higher in patients with stage IV than in those with Stages I–III lung cancer (Figure S2). A report indicated that cancer stages were related to CRP level and not to PCT level [43]. In the absence of a bacterial infection, nonspecific elevation of PCT level can occur in situations of massive stress [13, 63]. These inconsistent findings point to the need for further research in patients with lung cancer with no signs of infection.

Although the usefulness for diagnosis of bacterial infections and outcome assessment in patients with febrile nonneutropenic lung cancer has been partially confirmed, several limitations should be considered in the present study. First, given it was a retrospective study with a small number of patients, the effect of confounding variables cannot be ruled out [5]. Prospective studies involving a larger sample size are required to draw generally applicable conclusions. Second, inflammatory markers were measured only at the onset of fever, which could lead to overlooking any subsequent increases in infected patients [64]. The value of dynamic rather than absolute values should be considered in the assessment of febrile episodes in NNLCPs. Third, the high heterogeneity of enrolled patients or our inclusion and exclusion criteria could have affected our results. For example, only patients who did not have severe neutropenia (ANC < 500) were recruited while other neutropenic patients with ANC 500–1500 were indeed included. At the same time, mounting evidence suggests that high body mass index (BMI), obesity, diabetes, liver cirrhosis, and CKD were able to affect procalcitonin levels. However, in the current study the lung cancer patients complicated with obesity, diabetes, liver cirrhosis, and CKD were excluded, since these cases were rare. Besides, we did not analyze the relationship between BMI and procalcitonin, because body weights were affected by tumor occupying intensely and obviously and we failed to collect detailed information of patient's weight at the time of sample collecting. Finally, we relied on positive bacterial cultures from specimens to determine infections. However, there could have been false-negative bacterial culture results because of the sensitivity of the testing technique [5, 65]. Thus, we cannot completely rule out the possibility of infections in the TF group.

## 5. Conclusion

Overall, the present study demonstrated the superiority of PCT over CRP and NLR in differentiating infectious fever from TF. Additionally, markedly increased PCT level indicated a poorer treatment response and supported our conclusion that the PCT level can help predict the clinical outcome and cancer progression in NNLCPs. Further studies are needed to fully evaluate our findings.

## Figures and Tables

**Table 1:** Clinical and demographic characteristics of bacteria-infected group and TF group in NNLCPs with fever.

Characteristics	Bacteria-infected group (*N* = 311)	TF group (*N* = 277)
Age, median (range) (years)	65 (35, 87)	64 (28, 84)
Sex		
Male *n* (%)	246 (79.1)	189 (68.2)
Female *n* (%)	65 (20.9)	88 (31.8)
Pathological type		
AC *n* (%)	131 (42.1)	139 (50.2)
SCC *n* (%)	106 (34.1)	73 (26.4)
SCLC *n* (%)	54 (17.4)	56 (20.2)
Others *n* (%)	20 (6.4)	9 (3.2)
Tumor stage		
I *n* (%)	55 (17.7)	30 (10.8)
II *n* (%)	18 (5.8)	13 (4.7)
III *n* (%)	84 (27.0)	82 (29.6)
IV *n* (%)	135 (43.4)	133 (48.0)
Not available, EMR *n* (%)	19 (6.1)	19 (6.9)
Hematological parameters		
WBC median (range) (10^9^/L)	8.87 (0.89, 42.54)	5.57 (0.82, 29.36)
LYM median (range) (10^9^/L)	0.83 (0.05, 6.07)	1.11 (0.08, 8.14)
NEU median (range) (10^9^/L)	7.15 (0.73, 41.32)	3.82 (0.63, 23.21)
MON median (range) (10^9^/L)	0.62 (0.02, 3.83)	0.53 (0.04, 3.51)
RBC median (range) (10^12^/L)	3.87 (1.48, 5.50)	4.02 (1.54, 5.72)
Hb median (range) (g/L)	120 (46, 169)	124 (51, 172)
PLT median (range) (10^9^/L)	202 (2, 567)	217 (19, 772)
NLR median (range)	8.70 (0.83, 190.38)	3.63 (0.69, 39.00)
PLR median (range)	226.83 (2.38, 1480.00)	189.68 (10.58, 857.14)
LMR median (range)	1.45 (0.18, 62.50)	1.98 (0.31, 22.44)
Microorganism^a^		
Gram-positive bacteria *n* (%)	44 (13.8)	
*Staphylococcus aureus n* (%)	20 (6.3)	
*Streptococcus pneumoniae n* (%)	15 (4.7)	
Others *n* (%)	9 (2.8)	
Gram-negative bacteria *n* (%)	274 (86.2)	
*Klebsiella pneumoniae n* (%)	68 (21.4)	
*Haemophilus influenzae n* (%)	58 (18.2)	
*Pseudomonas aeruginosa n* (%)	44 (13.9)	
*Escherichia coli n* (%)	30 (9.4)	
*Acinetobacter baumannii n* (%)	17 (5.4)	
*Moraxella catarrhalis n* (%)	14 (4.4)	
Others *n* (%)	43 (13.5)	

^a^Seven patients were identified by MDI as having simultaneous infection with two types of bacteria. NNLCPs: nonneutropenic lung cancer patients; TF: tumor fever; AC: adenocarcinoma; SCC: squamous cell carcinoma; SCLC: small cell lung cancer; EMR: electronic medical records; WBC: white blood cell; LYM: lymphocyte; NEU: neutrophil; MON: monocyte; NLR: neutrophil-to-lymphocyte ratio; PLR: platelet-to-lymphocyte ratio; LMR: lymphocyte-to-monocyte ratio; MDI: microbiologically documented infection.

**Table 2:** Comparison of blood inflammatory markers between bacteria-infected subgroup A and TF subgroup A in NNLCPs.

Variable	Bacteria-infected subgroup A (*N* = 69)	TF subgroup A (*N* = 56)	*p* value Mann–Whitney
PCT median (range) (ng/mL)	0.24 (0.04, 18.62)	0.05 (0.02, 1.58)^a^	<0.0001
CRP median (range) (mg/L)	53.2 (0.6, 358.3)	6.3 (0.2, 89.4)	<0.0001
WBC median (range) (10^9^/L)	8.49 (1.1, 42.54)	5.24 (1.89, 15.82)	<0.0001
NEU median (range) (10^9^/L)	7.39 (0.73, 41.32)	3.56 (0.96, 13.94)	<0.0001
NLR median (range)	12.15 (1.03, 189.80)	3.27 (0.69, 39.00)	<0.0001

^a^Not to overestimate possible differences between the two groups, the values reported as <0.02 by the laboratory were noted as “0.02” for statistical analysis. NNLCPs: nonneutropenic lung cancer patients; TF: tumor fever; PCT: procalcitonin; CRP: C-reactive protein; WBC: white blood cell; NEU: neutrophil; NLR: neutrophil-to-lymphocyte ratio.

**Table 3:** Spearman correlations among the levels of PCT (ng/mL), CRP (mg/L), WBC (109/L), NEU (109/L), and NLR, measured within 48h of the onset of fever in bacteria-infected subgroup A.

	PCT	CRP	WBC	NEU	NLR
PCT					
*r*	1.00	0.746^*∗∗*^	0.222	0.285^*∗*^	0.511^*∗∗*^
95% CI		0.605 to 0.838	−0.029 to 0.448	0.045 to 0.499	0.293 to 0.685
*p*		<0.001	0.067	0.017	<0.001
CRP					
*r*		1.00	0.176	0.258^*∗*^	0.497^*∗∗*^
95% CI			−0.058 to 0.398	0.031 to 0.469	0.297 to 0.666
*p*			0.148	0.032	<0.001
WBC					
*r*			1.00	0.978^*∗∗*^	0.547^*∗∗*^
95% CI				0.955 to 0.986	0.378 to 0.673
*p*				<0.001	<0.001
NEU					
*r*				1.00	0.682^*∗∗*^
95% CI					0.559 to 0.776
*p*					<0.001
NLR					
*r*					1.00
95% CI					
*p*					

r Correlation coefficient calculated using Spearman's methods. TF: tumor fever; PCT: procalcitonin; CRP: C-reactive protein; WBC: white blood cell; NEU: neutrophil; NLR: neutrophil-to-lymphocyte ratio.

**Table 4:** The diagnostic performance comparison of PCT, CRP, WBC, NEU, and NLR in bacteria-infected subgroup A and TF subgroup A.

Diagnostic performance	PCT	CRP	WBC	NEU	NLR
AUC	0.874	0.855	0.729	0.761	0.792
95% CI	0.813–0.935	0.790–0.919	0.639–0.818	0.676–0.846	0.712–0.872
Cut-off value	0.105 ng/mL	12.2 mg/L	5.975 × 10^9^ L	6.245 × 10^9^ L	7.397
Sensitivity	79.7%	85.5%	78.3%	62.3%	66.7%
Specificity	80.4%	71.4%	64.3%	82.1%	87.5%
PPV	83.3%	78.7%	73.0%	81.1%	86.8%
NPV	76.3%	80.0%	70.6%	63.9%	68.1%
Youden index	0.601	0.569	0.425	0.445	0.542
*p* value	<0.001	<0.001	<0.001	<0.001	<0.001

TF: tumor fever; AUC, area under the ROC curve; PPV: positive predictive value; NPV: negative predictive value; PCT: procalcitonin; CRP: C-reactive protein; WBC: white blood cell; NEU: neutrophil; NLR: neutrophil-to-lymphocyte ratio.

**Table 5:** The treatment and clinical response in bacteria-infected subgroup A and TF subgroup A.

	Response to therapy				Total
	PD	SD	PR	unknown	
Bacteria-infected subgroup A (*N* = 69)					
Treatment					
Radiotherapy	13	11	1	5	30
Chemotherapy	3	12	0	0	15
Surgery					19
Others^a^					5
TF subgroup A (*N* = 56)					
Treatment					
Radiotherapy	2	13	4	8	27
Chemotherapy	0	10	3	8	21
Surgery					1
Others^a^					7

^a^Refer mainly to targeted combination, conservative treatment, or suspension therapy. TF: tumor fever; PD: progressive disease; SD: stable disease; PR: partial response.

**Table 6:** The levels of WBC, NEU, and NLR measured on presentation with microbiologically documented infections, in bacteria-infected subgroup B with high and low levels of PCT.

Variable	High PCT group (≥0.23 ng/mL) median (range)	Low PCT group (<0.23 ng/mL) median (range)	*p* value Mann–Whitney
PCT (ng/mL)	0.67 (0.23, 100)^a^	0.09 (0.03, 0.23)	<0.0001
WBC (10^9^/L)	11.61 (1.1, 42.54)	8.84 (2.82, 26.24)	0.0124
NEU (10^9^/L)	9.73 (0.73, 41.32)	6.905 (1.35, 22.65)	0.0011
NLR	14.42 (1.14, 190.38)	6.485 (1.03, 66.79)	<0.0001

^a^Not to overestimate possible differences between the two groups, the values reported as > 100 by the laboratory were noted as “100” for statistical analysis. PCT: procalcitonin; NEU: neutrophil; NLR: neutrophil-to-lymphocyte ratio.

**Table 7:** The treatment and clinical response in bacteria-infected subgroup B.

	Response to therapy				Total
	PD	SD	PR	unknown	
Treatment					191
Radiotherapy	20	23	2	17	62
Chemotherapy	14	21	3	12	50
Surgery					66
Others^a^					13

^a^Refer mainly to targeted combination, conservative treatment, or suspension therapy. PD: progressive disease; SD: stable disease; PR: partial response.

## Data Availability

The retrospective data used to support the findings of this study are included within the article.

## Abbreviations

NNLCPs: Nonneutropenic lung cancer patients
TF: Tumor fever
WBC: White blood cell
NEU: Neutrophil
CRP: C-reactive protein
NLR: Neutrophil-to-lymphocyte ratio
PCT: Procalcitonin
MDI: Microbiologically documented infection
ROC: Receiver operating characteristic
PD: Progressive disease
SD: Stable disease
PR: Partial response.

## Supplementary Materials

Supplementary Materials Figure S1: schematic of patients' recruitment and classification for study. TF: tumor fever; PCT: procalcitonin; CRP: C-reactive protein; NNLCPs: nonneutropenic lung cancer patients. Figure S2: Comparison of PCT and CRP levels and NLR in patients with stage I to III and stage IV in TF subgroup A. There was no significant difference in PCT (A) and CRP (B) levels and NLR (C) between patients with stage IV (*n* = 31) and those with I to III (*n* = 21) in TF subgroup A. ns: no significance; TF: tumor fever; PCT: procalcitonin; CRP: C-reactive protein; NLR: neutrophil-to-lymphocyte ratio. Supplementary Table 1: the levels of PCT (ng/mL), CRP (mg/L), WBC (109/L), NEU (109/L) and NLR, in microbiologically documented infections, distinguishing Gram-positive and Gram-negative.
