# Supplementary material for: Diagnostic Accuracy of Procalcitonin, Neutrophil-to-Lymphocyte Ratio, and C-Reactive Protein in Detection of Bacterial Infections and Prediction of Outcome in Nonneutropenic Febrile Patients with Lung Malignancy
Source: J Oncol. 2020 Aug 25;2020:2192378. doi: 10.1155/2020/2192378 (PMC7468665; doi:10.1155/2020/2192378)
Supplement: Supplementary Materials — Figure S1: schematic of patients' recruitment and classification for study. TF: tumor fever; PCT: procalcitonin; CRP: C-reactive protein; NNLCPs: nonneutropenic lung cancer patients. Figure S2: Comparison of PCT and CRP levels and NLR in patients with stage I to III and stage IV in TF subgroup A. There was no significant difference in PCT (A) and CRP (B) levels and NLR (C) between patients with stage IV (n = 31) and those with I to III (n = 21) in TF subgroup A. ns: no significance; TF: tumor fever; PCT: procalcitonin; CRP: C-reactive protein; NLR: neutrophil-to-lymphocyte ratio. Supplementary Table 1: the levels of PCT (ng/mL), CRP (mg/L), WBC (109/L), NEU (109/L) and NLR, in microbiologically documented infections, distinguishing Gram-positive and Gram-negative. [file 2192378.f1.docx]

**Supplementary Figures**

**
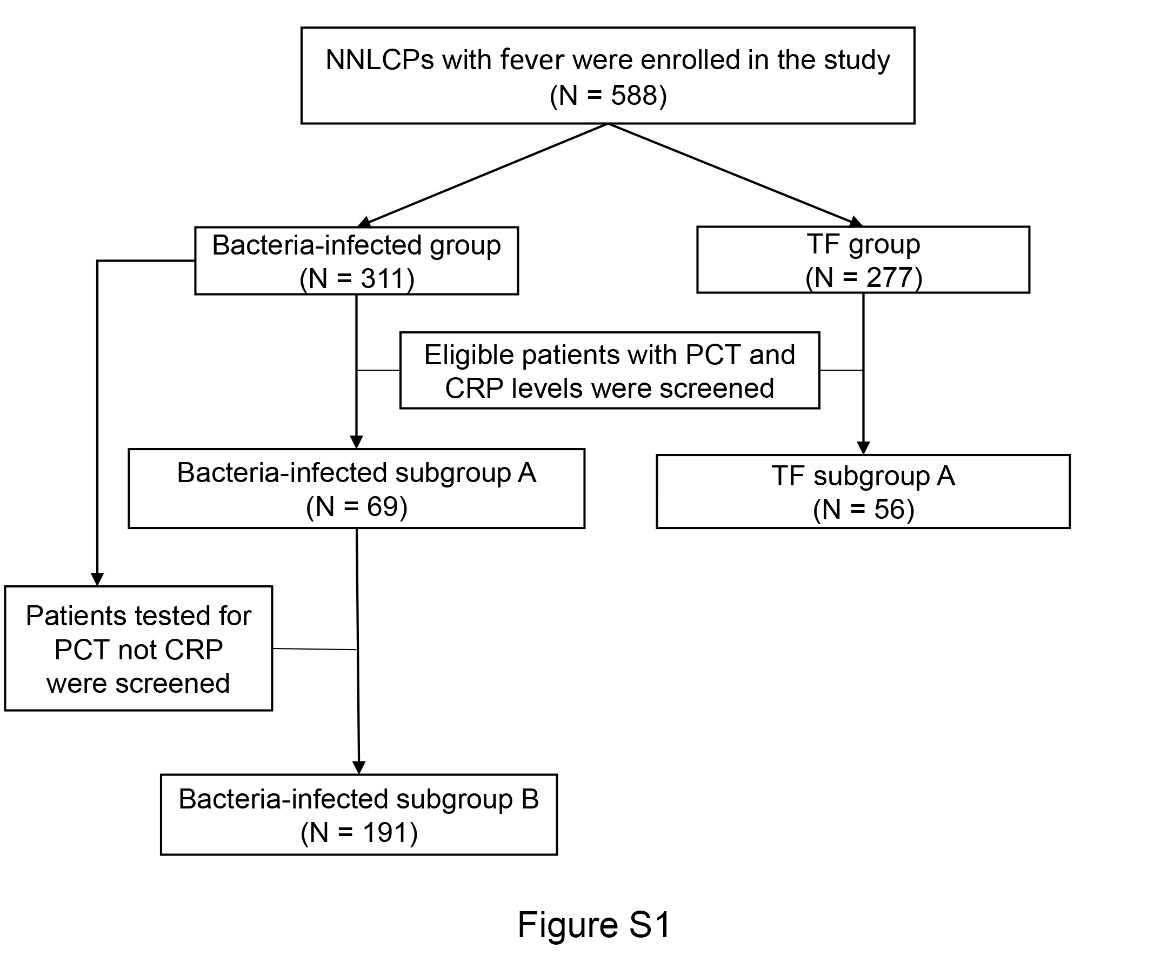
**

**Figure S1: Schematic of patients recruitment and classification for study.** TF, tumor fever; PCT, procalcitonin; CRP, C-reactive protein; NNLCPs, non-neutropenic lung cancer patients.

**
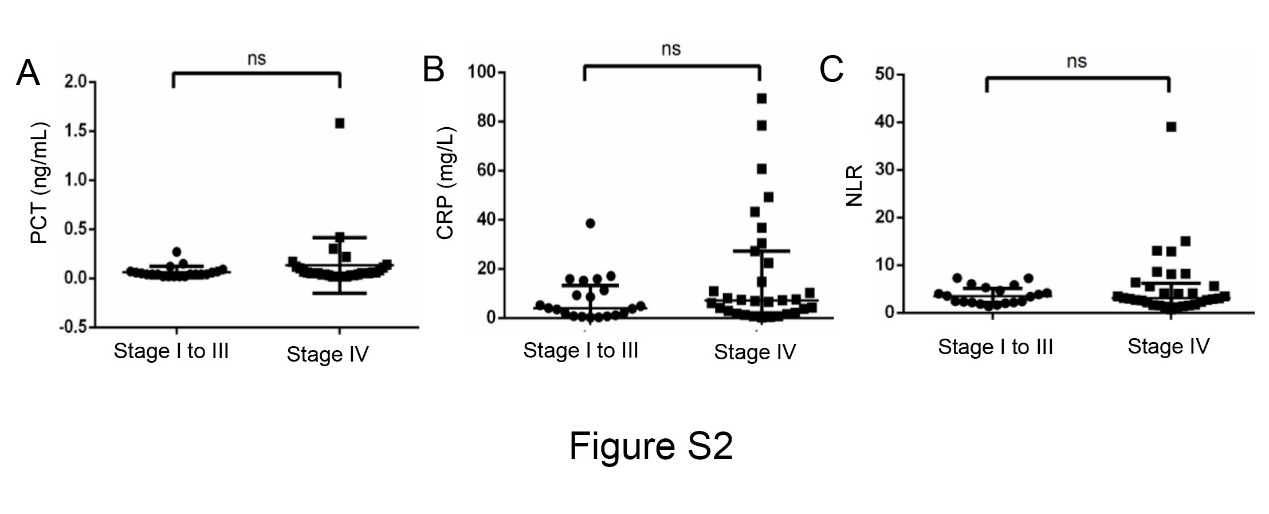
**

**Figure S2: Comparasion of PCT and CRP levels and NLR in patients with stage I to III and stage IV in TF subgroup A.** There was no significant difference in PCT(A) and CRP (B) levels and NLR (C) between patients with stage IV (n=31) and those with I to III (n=21) in TF subgroup A. ns, no significance; TF, tumor fever; PCT, procalcitonin; CRP, C-reactive protein; NLR, neutrophil-to-lymphocyte ratio.

**Supplementary Table**

**Supplementary table1．The levels of PCT (ng/mL), CRP (mg/L), WBC (10^9^/L), NEU (10^9^/L) and NLR, in microbiologically documented infections, distinguishing Gram-positive and Gram-negative.**

|  | **Gram-positive bacteria^a^** | | | **Gram-negative bacteria^a^** | | ***P*-value**  **Mann-Whitney** |
| --- | --- | --- | --- | --- | --- | --- |
|  | *N* | Median (range) | *N* | | Median (range) |  |
| **PCT** | 29 | 0.27 (0.04, 100)^b^ | 165 | | 0.23 (0.03, 100)^b^ | 0.7664 |
| **CRP** | 11 | 43.8 (4.3, 257.1) | 58 | | 55.6 (0.6, 358.3) | 0.6626 |
| **WBC** | 44 | 8.52 (2.06, 32.15) | 275 | | 8.925 (0.89, 42.54) | 0.5098 |
| **NEU** | 44 | 7.125 (1.35, 26.02) | 275 | | 7.175 (0.73, 41.32) | 0.8289 |
| **NLR** | 44 | 10.95 (1.04, 189.8) | 275 | | 8.518 (0.83, 190.38) | 0.1316 |

a Seven patients were identified by MDI as having simultaneous infection with two types of bacteria.

b Not to overestimate possible differences between the two groups, the values reported as > 100 by the laboratory were noted as “100” for statistical analysis.

PCT, procalcitonin; CRP, C-reactive protein; NEU, neutrophil; NLR, neutrophil-to-lymphocyte ratio; MDI, microbiologically documented infection.
